# Editorial: Potential of the plant rhizomicrobiome for bioremediation of contaminants in agroecosystems

**DOI:** 10.3389/fpls.2024.1397360

**Published:** 2024-04-22

**Authors:** Geeta Bhandari, Saurabh Gangola, Pankaj Bhatt, Mohd Rafatullah

**Affiliations:** ^1^ Himalayan School of Biosciences, Swami Rama Himalayan University, Dehradun, Uttarakhand, India; ^2^ School of Agriculture, Graphic Era Hill University, Bhimtal, Uttarakhand, India; ^3^ Integrative Microbiology Research Centre, South China Agricultural University, Guangzhou, China; ^4^ Environmental Technology Division, School of Industrial Technology, Universiti Sains Malaysia, Penang, George Town, Malaysia

**Keywords:** rhizomicrobiome, agro-ecosystems, pollutants, microbes, PGPR

The plant rhizomicrobiome comprises of complex communities of symbiotic rhizobacteria, mycorrhizal fungi, protozoans, and free-living microbes. The rhizomicrobiome is vital for maintaining the biogeochemical balance of soil, nutrient cycling, soil health, plant performance, disease suppression, stress tolerance and carbon sequestration (Alotaibi et al., 2021). In the past decade, research has unveiled another remarkable aspect of this relationship: ability of rhizomicrobiomes to remediate contaminated agro-ecosystems. Potential rhizobacterial communities accelerate the contaminant degradation processes in a plant assisted co-metabolism process (Su et al., 2022; GAngola et al., 2023). The rhizospheric zone can tolerate contaminant stress, especially xenobiotics and pesticides, primarily due to microbial functions associated with metabolic biodegradation of contaminants (Lü et al., 2023). The contaminant degradation pathways either fully or partially detoxify the soil or reduce the amount and toxicity of contaminants. Moreover the microbial genes encoding degradative enzymes capable of contaminant degradation enrich the rhizosphere (Huang et al., 2023). Thus, understanding the functionalities of rhizomicrobiome communities is essential for the development of sustainable remediation methods. This editorial reviews the current developments in this field, drawing insights from articles published in this Research Topic. The present Research Topic comprises of three research and one review article.

## Harnessing microbial partnerships for bioremediation

The plant growth-promoting characteristics of *Bacillus cereus* and their impact on the potential for phytoremediation in metal-contaminated soils are insightfully explained by Narayanan et al. The authors have evaluated the synergistic interaction between *Bacillus cereus NDRMN001* and *Cajanus cajan* (L.) as a long-term strategy for the treatment of metal-contaminated soils. The metal-tolerant strain *Bacillus cereus NDRMN001* was reported to exhibit remarkable plant growth promoting activities and significantly enhance the phytoremediation capability of *Cajanus cajan* for metal-polluted soil. By elucidating the mechanisms underlying this phenomenon, this research lays the foundation for developing sustainable bioremediation strategies that harness the power of beneficial microbes. Parallel to this, Han et al. investigated the role of plant genes, including LbKAT3, in the uptake of nutrients from the rhizosphere. Although it is well known that mycorrhizal associations help plants in nutrient absorption, its potential for bioremediation has just lately come into question. The role of AM fungus, *Rhizophagus irregularis* on potassium uptake by *Lycium barbarum* was determined. The results imply that inoculating the AM fungi with simultaneous application of potassium caused *L. barbarum* to have higher dry weight, potassium, and phosphorus contents. The enhanced expression of LbKAT3 and AQP genes in the plant suggested its role in potassium uptake as well as the transport of phosphorus and water from AM fungi to the host plant. This mutualistic interaction has the potential to improve the sustainability of agricultural systems and restore nutrient-deficient soils.

## Understanding plant responses to contaminants

Marabesi et al. have examined the complex interplay between environmental pollutants and plant gene expression. The impact of cadmium (Cd) uptake on the physiology, growth, and gene expression of *Cannabis sativa* L. (hemp) plants was determined. Stunted growth, decreased photochemical efficiency, and early senescence were observed in the presence of high Cd levels, and thus suggested Cd toxicity in hemp plants. Metal associated transporter gene expression was observed under Cd stress. This study can play a crucial role in creating customized bioremediation plans for different pollutants and plant species.

## Innovative bioremediation techniques and future prospects

Khan et al. suggest the application of microbial bioformulations for environmentally friendly farming practices. Utilizing the mutually beneficial relationships between plants and microbes can improve agricultural productivity, soil fertility, and resistance to environmental stressors. Furthermore, by encouraging the development of microbial communities that can degrade pollutants and improve nutrient cycling, microbial bioformulations have the potential to increase the bioremediation capability of agroecosystems. The main focus of this work is on the investigation of microbial bioformulations and their carrier materials. It offers information on selection criteria, bioformulation development, best practices, and precautions for different types of agricultural land. Another prime focus of this work is to assess the potential of bioformulations for supporting plant growth and pathogen defense mechanisms, taking into consideration biosafety issues. “Tailored rhizoremediation formulations” capable of adapting well to stressed environmental circumstances can be developed as efficient remediation technologies.

The intricate process of microbial degradation relies on the microbial diversity in individual habitats and is greatly influenced by nutrient cycles and stress response processes (GAngola et al., 2022; Rodríguez et al., 2022). In order to determine which chemical variables and microbial diversity metrics can predict chemical degradation, it is necessary to comprehend the relationship between the composition of the microbial community, its potential for degradation, growth regulators, resistance, and metabolic capabilities of microbes to deal with xenobiotics. Furthermore, bioengineering of synthetic microbial communities is a promising approach for improving plant development and contaminant degradation. Advances in next generation sequencing, metagenomics, transcriptomics, proteomics, genome editing tools, and bioinformatics are improving our understanding of the relationships between plants and microbes, which can assist in development of efficient and economically sustainable remediation strategies.

## Conclusion

The articles in this Research Topic highlight the plant rhizomicrobiome’s enormous potential for bioremediation of contaminated agroecosystems. By harnessing microbial interactions, comprehending plants’ responses to contaminated soil and developing novel remediation strategies, scientists are paving the way towards sustainable agro-ecosystems. Furthermore, interdisciplinary research and continued efforts will be significant in transforming these discoveries into solutions that address the challenges of environmental contamination.

## Author contributions

GB: Writing – original draft, Writing – review & editing. SG: Writing – review & editing. PB: Writing – review & editing. MR: Writing – review & editing.
